# Editorial: NeuroCOVID. Insights into the clinical manifestations and pathophysiology

**DOI:** 10.3389/fneur.2025.1561314

**Published:** 2025-02-11

**Authors:** Alina González-Quevedo, Pedro A. Valdés Sosa, Calixto Machado Curbelo, Joel Gutiérrez Gil

**Affiliations:** ^1^Department of Neurobiology, Institute of Neurology and Neurosurgery, Havana, Cuba; ^2^Medical Sciences University of Havana, Havana, Cuba; ^3^Clinical Hospital of Chengdu Brain Science Institute, University of Electronic Science and Technology of China, Chengdu, China; ^4^Cuban Neuroscience Center, Havana, Cuba; ^5^Department of Neurophysiology, Institute of Neurology and Neurosurgery, Havana, Cuba

**Keywords:** COVID-19, cytokine storm, neuroinvasion, neurological manifestations, long COVID

Coronavirus Disease 2019 (COVID-19), caused by Severe Acute Respiratory Syndrome Coronavirus 2 (SARS-CoV-2), led to a global pandemic that placed the world in the worst health situation of the last century. It is known to cause multi-organ dysfunction during the acute phase and some patients experience prolonged symptoms. In both instances the nervous system has been reported to be affected. Neurological complications were identified for the first time in 36.4% of hospitalized patients in Wuhan (1). From then on, subsequent different variants of COVID 19 with varying frequency of neurological complications have appeared, rendering the comparisons between different subtypes difficult. For example, the Delta variant is known to enhance the susceptibility to develop ischemic stroke, epilepsy, seizures, and cognitive impairments in infected individuals (2).

Furthermore, not all studies analyze neurological manifestations in the same manner. Some consider neurological signs and symptoms, others focus mainly on neurological disorders and some combine symptoms and diseases. These neurological manifestations can be roughly divided into two categories - those occurring during the acute phase of the infection, generally associated with the direct invasion of central or peripheral nervous tissues by the virus or due to severe systemic or neuroinflammatory responses (cytokine storm), and the post-viral complications (3).

This editorial approaches the analysis of the 8 articles published in the Research Topic, which are focused on COVID-19 associated acute and post-acute neurological manifestations, and extends to a broader analysis of the general situation that NeuroCOVID imposes for research purposes and public health policies (4).

The frequency of neurological manifestations is high in COVID-19, especially in hospitalized patients; although from the two studies included in this Research Topic it is not possible to estimate the incidence, because only adult patients who developed new-onset neurological symptoms during hospitalization were included. Hanganu et al. evaluated 115 patients, reporting that central nervous system (CNS) manifestations were more frequent than the involvement of peripheral nervous system (PNS), and that they were independently associated with older age, disease severity, ischemic heart disease, and increased D-dimers. COVID-19-associated encephalopathy was the most common CNS manifestation, but neurovascular events were also important. On the other hand, Mazraeh et al. focused mainly on clinical symptoms, describing a wide range that include CNS manifestations such as headaches, dizziness, altered mental state, and disorientation, as well as PNS symptoms involving impairment of taste and smell, and musculoskeletal issues. In children neurological complications can acquire very severe life-threatening manifestations, as revealed by Zhang et al. in 4 children who developed acute necrotizing encephalopathy after infection with the Omicron BF.7.14 coronavirus, which had not been previously described with other variants. Previously, a rare delayed hyperinflammatory response to SARS-CoV-2 infection (multisystem inflammatory syndrome) had been described in children, especially prevalent with the Alpha variant (2).

Focusing on the PNS, Yu et al. centered their investigation on how COVID-19 significantly increased the incidence and severity of peripheral facial nerve paralysis (PFNP) throughout the pandemic years. On a similar basis, Tereshko et al. explored the involvement of the PNS in 30 patients with previous SARS-CoV-2 infection and normal sensory nerve conduction studies, 3 months after the disease. Employing the Semmes–Weinstein monofilament test, they demonstrated impaired tactile sensation of A-beta nerve fibers, suggesting post-COVID subclinical damage to sensory fibers.

The high prevalence of brain damage which has been reported as a consequence of SARS in COVID-19 does not seem to be related specifically to SARS-CoV-2 infection. According to the neuropathological findings of Humayun et al., the overall frequency of acute brain injury in COVID-19 and non-COVID-19 patients with SARS, is not related with the specific etiology of lung injury. The underlying culprits seem to be severe systemic inflammation and hypoxemia, taking part in an inexorably damaging brain-lung crosstalk. We should keep in mind that mild respiratory symptoms, like cough and dyspnea, can worsen into hypoxemia, leading to a cytokine storm, which can cause significant brain damage, frequently requiring intensive care. Early therapeutic interventions, including non-invasive ventilation methods, are recommended to prevent hypoxemia and curb the progression of the cytokine storm (5).

Two studies in this Research Topic engaged in the investigation of cognitive performance, which has been reported to be impaired post-COVID-19. Hotz et al. showed notable cognitive deficits after SARS-CoV-2 infection, especially in the domain attention. The treatment strategies were inconclusive and spontaneous remission of cognitive impairment could not be excluded. On the other hand, Chaganti et al. observed that cognitive impairment was associated with loss of white matter integrity, possibly mediated by blood-brain barrier breakdown and related glutamatergic excitotoxicity at 3 months, which improved at 12 months.

Summarizing, COVID-19 may lead to a wide variety of neurological manifestations, covering a clinical spectrum which can extend from relatively mild symptoms (headache, dizziness, myalgia, smell and taste impairment) to serious neurological complications (stroke, venous sinus thrombosis, encephalomyelitis and Guillain Barre syndrome, among others).

Almost 5 years after the first COVID-19 cases were detected, the field of neuro-COVID research is still in its early stages and much is yet to be learned about the long-term effects of COVID-19 on the nervous system. Many patients exhibit complex long-lasting brain fog, corresponding with long COVID effects on the brain, which manifest from psychological to functional neurological disorders (confusion, fatigue, short-term memory loss, dizziness, distraction, and reduced mental acuity), that significantly impair daily life activities. Very recently this condition has been associated with the accumulation of persistent viral spike protein in the skull, meninges and brain, together with sustained systemic inflammation, long after complete viral clearance (6). Furthermore, SARS-CoV-2 can trigger or exacerbate symptoms associated with neurodegeneration such as Parkinson' s disease, Alzheimer, and Multiple Sclerosis (3).

These and other studies are opening new avenues to optimize prevention, diagnostic tools and therapeutic strategies targeting acute and long-term neurologic complications of COVID-19. As the years pass, investigators are gaining more knowledge on the long-term effects and pathophysiology of COVID-19 on the nervous system. This is a challenging clinical issue due to the great number of patients affected by SARS-CoV-2 infection throughout the world.
